# Do Androids Dream of Lived Experience? Navigating Risks, Uncertainty, and Best Practices Around Generative AI in Patient and Public Involvement and Engagement

**DOI:** 10.2196/96621

**Published:** 2026-09-30

**Authors:** William Lammons, Selina Wallis, Yuncong Liu, Lorraine Cezair-Phillip

**Affiliations:** 1 Primary Care and Population Health, University College London, 1-19 Torrington Place, London, England, WC1E 7HB, United Kingdom, 44 20 3108 5599; 2 NIHR ARC North Thames, London, England, United Kingdom; 3 NIHR ARC North West Coast, Liverpool, England, United Kingdom; 4 Department of Health Services Research, University of Liverpool, Liverpool, England, United Kingdom

**Keywords:** patient and public involvement and engagement, artificial intelligence, lived experience, AI chatbots, PPIE

## Abstract

In this viewpoint paper, we, as professionals working in patient and public involvement and engagement (PPIE), argue that the use of generative AI poses risks to the place of lived experience in PPIE because of its ability to generate outputs that can potentially eclipse those of people with lived experience. Generative AI appears to offer solutions to the National Health Service (NHS) and health research in an era of change and uncertainty—overstretched health infrastructure, unclear leadership, burned-out staff, and cuts and redundancies. NHS leaders are also pursuing the integration of generative AI into the NHS, in part to realize its “analogue to digital” plans. But those solutions come with commensurate risks. In this context, we have authored this viewpoint, aiming to (1) explain the risks that generative AI poses to those working with lived experience in PPIE contexts (distorted and biased outputs, contributing to inequalities, minimizing or replacing patient voices, and limiting learning); (2) clarify the positive opportunities that generative AI offers to PPIE (speeding up diagnostics, accessibility accommodations); and (3) provide a checklist that gives professionals clear pragmatic advice on using generative AI ethically in PPIE.

## Introduction

### Our Viewpoint

The use of generative AI threatens the place of lived experience in patient and public involvement and engagement (PPIE) because its outputs can (1) be distorted and biased, (2) be based on unrepresentative training data and contribute to inequalities, (3) minimize or replace patient voices, and (4) reduce learning that emerges from PPIE activities. With the advent of the generative AI platform Panelyze, Steele et al [1] demonstrate that a research team can now use AI to generate personas with which they hold simulated discussions to produce content such as plain English summaries, all without the collaboration of real living patients. We, a group of 3 PPIE professionals and a member of the public, have written this viewpoint to discuss the ethical imperative of lived experience in PPIE, in response to the current digitization of UK public services and the rising visibility and prevalence of platforms like Panelyze and ChatGPT [2,3].

We write to all interested parties, including patient communities, researchers, PPIE professionals, health care professionals, patient advocates, and anyone working, volunteering, or advocating in spaces around lived experience. We aim not to denounce AI but to argue that large language model (LLM)–based generative AI, including and beyond Panelyze, poses risks to PPIE, collaborative research, and lived experience. LLMs, like ChatGPT, Co-Pilot, and Claude, are computer software programs that are “fed” or given large amounts of text so they can “learn” or identify patterns in language and return fluent, plausible, comprehensible language that mimics human speech [4]. They are designed to receive written or spoken messages and return comprehensible messages, and in the case of Panelyze, they hold the potential to reduce the place of patients and the public in collaborative research.

In this piece, we aim to help establish best practices for using generative AI, including chatbots like ChatGPT, in spaces where professionals contend with and work with the lived experiences of patients and members of the public, not only for current practice but also for the directions that future developments may take. To put it plainly, we do not want the outputs of generative AI to replace, in part or in whole, the voices and contributions of patients and members of the public with lived experiences.

The term “lived experience” comes from phenomenology, a philosophical tradition based on the understanding that “only those who have experienced phenomena can communicate them to the outside world” in a way that is more authentic than second-hand knowledge or even scientific inquiry [5]. The recognition that lived experience is a valuable source of knowledge arose through civil rights and liberation struggles of minoritized groups, that is, of racialized and disabled people, and those with a mental health diagnosis [6,7]. Currently, the term is used to signal the value of first-hand knowledge of a particular condition (such as cancer or depression) or treatment, alongside scientific knowledge, in the context of PPI in health service development and research [8-10].

This paper illustrates our own thoughts and reflections as individuals working in collaborative PPIE research spaces between members of the public with lived experiences and researchers. It does not reflect any official statements of our current or previous host organizations, that is, the Public Involvement Community (PIC), the National Institute for Health and Care Research (NIHR), the NIHR Applied Research Collaborations (ARCs), or the National Health Service (NHS). These thoughts first emerged from a discussion with PIC members, a collective of PPIE professionals working across various NIHR ARCs, research implementation and collaboration networks within the research arm of the NHS, and the NIHR. We noticed a sudden interest in the use of generative AI in PPIE in the summer of 2025 [11,12], and we wrote this paper following our discussion about how best to respond to this new technology, while holding space for the opportunities and risks generative AI presents for patients and lived experiences in PPIE.

### Setting Context

#### The NHS and Health in the United Kingdom

Currently, the United Kingdom’s health landscape faces a combination of funding issues and efficiency drives through organizational restructuring and consolidation [13] as well as extensive staff redundancies [14], in part to address growing NHS waiting lists [15,16] and clinician shortages [3,17]. Meanwhile, UK universities face a funding crisis in which most have budget deficits or reduced surpluses, similarly leading to efficiency and cost-cutting drives, such as large-scale redundancies [18,19]. AI has been positioned as a solution to these economic pressures by amplifying efficiency and productivity. For instance, generative AI chatbots such as Claude, Co-Pilot, or ChatGPT can be used to reduce administrative burdens, and AI analytical techniques like machine learning and deep learning have been found to improve diagnostic speed and save clinicians’ time by analyzing medical data and scans [2].

#### Analogue to Digital

The NHS’s 10-year plan focuses heavily on a move from analogue to digital systems and record keeping [3]; venture capital, software, and tech companies are spending hundreds of billions in AI investment and development [20]; and health infrastructure is rapidly adopting AI tools such as generative AI chatbots [2,21]. In the NHS and the NIHR, AI systems are analyzing medical images [22], detecting and supporting the treatment of diseases at earlier stages [23], supporting staff administrative work [24], facilitating clinician notetaking [21], and transcribing recordings of PPIE activities [25]. The data analysis and AI tech company Palantir has also had ongoing contracts worth hundreds of millions of pounds with the UK government since 2023, relating to health data analysis and digitizing public health services [26-28]. Palantir has even recently been granted “unlimited access” to “NHS patient data” [29].

The drive to bring AI technologies into the United Kingdom’s health care infrastructure and the needs presented by the NHS are not without their challenges, and arguably the speed of adoption and technological advances pose security threats. As AI is increasingly integrated into critical infrastructure, malicious actors can potentially leverage these technologies to scale cyberattacks and exploit systems [30,31]. Other health and research-related issues include a lack of leadership, limited clarity, difficulties with implementation, and ethical issues [2,25,27-29,32,33]. John Uttley [32], Innovation Director and Senior Information Risk Owner (SIRO) of the NHS Midlands and Lancashire, has recently pointed out the lack of a “shared framework for AI governance” across NHS regions. He argues that those working in the NIHR and the NHS need clear, collective positions across the health and research ecosystem [32]. Without this, ad hoc approaches may emerge and undermine best practices by creating variations in practices across the country [32]. Patil and Stavropoulou [2] have shown in a complementary way that despite the techno-optimism, financial support, and speed driving AI technologies’ integration into the NHS, many questions remain about how to actually bring AI into practice in the NHS, thereby creating hurdles to their success.

Finally, in recent news, Palantir’s relationship with the NHS, especially regarding access to patient health data, has faced strong criticism. For example, the House of Commons has called for an end to the relationship, largely because of Palantir’s role in supporting the US Immigration and Customs Enforcement (ICE) in an overwhelming push to arrest suspected undocumented immigrants [27]. Thus, the push to bring AI into the NHS, despite its benefits, faces criticism and unanswered questions about practicality and applications.

#### PPIE in an Era of Change and Uncertainty

PPIE sits in a similar and parallel position of uncertainty to the NHS and UK universities—overwhelmed staff, cuts, AI opportunities, and unclear leadership [34-36]. For example, in April 2025, the NIHR opened a contract for the Maximising Impact and Public Partnerships (MIPP) Coordinating Centre, the first formal and centralized PPIE entity since INVOLVE ended in 2020 [34,37]. However, the NIHR suddenly ended MIPP in January 2026 with little explanation, creating confusion, and missing the opportunity to unite and guide best practices [34,37].

Patients and the public themselves hold nuanced and complex views regarding AI. A recent qualitative evidence synthesis of 12 studies found that various members of the public perceived both positive and negative potentials of AI in clinical practice [38]. Positive perceptions included data storage, information efficiency, and real-time health data monitoring, whereas negative perceptions included concerns about data protection, privacy, reliability, overreliance, and economic impact [38]. Since that publication, other authors have noted patients’ interest in AI supporting diagnostic pathways, improving health care systems, and improving care quality [39,40]. Several studies report persistent negative perceptions and concerns about data security and trust in AI [39-42].

### Aims

We have composed this viewpoint piece to establish what we believe are the risks and suggest best practices around generative AI and PPIE by (1) explaining the risks that LLMs pose to the voices of those with lived experience, (2) clarifying the opportunities that generative AI offers for PPIE in health research, and (3) giving professionals clear, pragmatic advice on how to use generative AI to preserve the place of people with lived experience in collaborative research.

We state the essential role of people with lived experience in health care, health care research, and PPIE in the emerging world of AI. In this piece, we clarify both that AI can help those with lived experience and professionals working in lived experience spaces and that, simultaneously, those with lived experience cannot be replaced by generative AI. We must maintain spaces for patient voices as leaders and colleagues, and as we ourselves begin to use generative AI chatbots to fill gaps and navigate challenges. We join other colleagues who have recently made similar arguments about maintaining spaces for human voices amid the rise of AI [43,44]. We have included a list, labeled as “signed,” of those who read, reviewed, and supported the final version of this paper’s argument but who did not participate in the writing process. Our choice to include a “signed” list was based on other timely papers that critique AI in the research world and use lists of signers to show broad support for such critiques [43].

## The Risks of Generative AI

### Defining Risks

The use of generative AI and chatbots presents risks to PPIE and lived experience in health care research. On reflection, we currently find that the risks of generative AI include (1) distortions and biases, (2) unrepresentative training data and contributions to inequalities, (3) minimizing or replacing patient voices, and (4) reductions in learning that emerges from PPIE activities. As this is a viewpoint paper, not a research or review study, our selection of these 4 categories of risk does not reflect a systematic approach, an exhaustive list of risks, or a definitive categorization or grouping of themed risks.

### Risk 1: Distorted and Biased Outputs

One of the clearest issues with using chatbots’ outputs as proxies for, or as equally valid to, lived experiences is the unique set of distortions produced in their attempts to sound and appear more human. Some scholars have termed these phenomena “quasi-human” [45-48], including, but not limited to, hallucinations, overconfidence, and sycophancy.

All generative AIs currently “hallucinate” or invent text that is factually inaccurate, for example, invented quotations, incorrect information, nonexistent bibliographic citations stitched together from actual ones, or inaccurate statistics [48-51]. For example, Google’s AI summary accompanying its search has been noted, as of January 2026, for providing factually inaccurate health information to users which endangered their health [52,53]. However, the risk varies by model, task, prompt, and whether and how retrieval or checking tools are used.

An important part of “quasi-humanness” is chatbots’ ability to present interactive language that connects with human users [48]. This can take the form of outputs that sound confident, authoritatively correct, or accurate, regardless of whether they actually are [49,50,54]. It can also be found in “sycophantic” responses or chatbot outputs that play to users’ anticipations, that is, “Yes, William, this is an excellent study; it will likely get published,” or “This is a very competitive application” [48,55,56].

Importantly, hallucinations, overconfidence, and sycophancy are not simply random errors but predictable risks of current LLM chatbots. Because they generate likely text rather than inherently verifying truth, they can produce confident inaccuracies [49,50,54,57,58].

### Risk 2: Training Data and Inequalities

Consulting LLM-based generative AI chatbots to create outputs to be treated as proxies for lived experiences is a category error and a threat to validity. These technologies cannot provide lived experiences; they generate text from patterns in their training data. When used in this way, they risk flattening differences between human cultures, contexts, and forms of knowledge. This risk is heightened because the data used to train many LLMs may underrepresent, distort, or marginalize the experiences of marginalized groups or minoritized people [41,42,59-61]. This is exacerbated by the lack of transparency around LLM development and training data, often called the “black box” nature of AI [41,62,63].

One clear and immediate consequence is the reproduction and reinforcement of existing biases, stereotypes, and racist perspectives against minoritized peoples, both subtly and overtly [64-70]. Recent high-profile examples show that even well-funded LLMs can generate racist, antisemitic, sexist, or otherwise discriminatory outputs, which can also intensify racial inequalities [64-70]. In July 2025, for example, Twitter’s (subsequently rebranded X) AI chatbot, Grok (now xAI), declared itself “Mecha Hitler” and generated antisemitic responses [64-70]. Importantly, LLMs did not invent biases, minoritization, racism, or stereotyping. However, when deployed at scale, they can legitimize and amplify these harms, particularly when their outputs are treated as authoritative or representative. Voice4Change England’s work on “Equity by Design,” together with wider research on AI and health inequalities, highlights the relevance of these risks in the United Kingdom’s health contexts where biased data, opaque model development, and insufficient governance may worsen existing health inequalities and racial disparities [41,63,70,71].

The physical infrastructure required to develop and operate effective generative AI also raises environmental justice concerns. Data centers require substantial electricity, water, land, and cooling infrastructure, and their expansion is increasingly affecting rural and disadvantaged communities. In the United States, this has become visible, especially in parts of Mississippi and the wider American South, where AI-related data centers have prompted concerns about fossil fuel usage, air pollution, noise, water demand, local infrastructure pressure, and community consent [72-74]. These concerns are particularly significant where facilities are sited in areas already shaped by poverty, racial inequality, and existing health disparities [72-74]. The issue is not limited to the United States. In the United Kingdom, such as London’s Brick Lane, data centers are also raising questions about energy demand, water use, community impact, planning infrastructure, and environmental governance [75]. Where data centers depend on fossil fuels, back-up diesel generation, or strained water systems, they may contribute to environmental conditions associated with respiratory, noise-related, and other health burdens. In this sense, generative AI not only represents lived experience poorly at the level of language and data, but its material infrastructure may also create or intensify lived experiences of environmental and health inequality [74,76,77].

Importantly, issues like bias, hallucinations, opacity, overconfidence, data retention, and inequality are features and risks of current systems in LLMs and generative AI. These risks may change over time, and some may be reduced through technical, regulatory, and participatory approaches. For example, “retrieval-augmented generation” (RAG) models connect an LLM to selected external sources, such as clinical guidelines or other curated evidence, so that outputs can be grounded more directly in retrieved documents rather than relying only on the model’s internal statistical patterns [78]. Related work on “explainable” “transparent AI” and “white box” or “glass box” LLMs seeks to make data use, decision-making, functioning, development, and evaluation of AI models more visible to users [62,78,79]. The AI-MULTIPLY project offers an example of applying PPIE as a participatory approach to AI development [41], and Banerjee et al [42] provided a helpful framework for building public trust in AI through participatory data science.

However, even if these developments lead to more inclusive, equitable, and reliable LLM-based generative AI chatbots, relying on these technologies instead of collaborating with real people with lived experiences would still weaken PPIE in 2 important ways. First, it would displace the people whose experiences, relationships, and forms of knowledge should be central to involvement. Second, it would reduce the learning that researchers, practitioners, and staff gain through direct dialogue, shared reflection, and relationship building. PPIE is not only a mechanism for gathering views, it is also a relational process through which research questions, assumptions, and practices can be challenged and changed.

### Risk 3: Minimizing or Replacing Patients’ Voices

Steele et al [1] proposed Panelyze platform represents one of the clearest early attempts to use generative AI to simulate PPIE. The authors identify familiar and important limitations in current PPIE practice, including overrepresentation by “majority demographics” and the absence of “marginalized voices” as issues that PPIE faces, which need to be resolved. Their proposed response is Panelyze, an LLM-based system that draws on Anthropic’s Claude chatbot to generate synthetic personas and panels intended “to embody underrepresented demographics” [1]. The authors describe the system’s “primary innovation” as “its ability to surface qualitative ‘lived experience’; insights in silico...” [1]. Although Steele et al [1] repeatedly frame Panelyze as an augmentation tool or first pass process intended to improve later human PPIE work, rather than replace it, the paper’s language raises important conceptual and ethical concerns. In particular, its descriptions of synthetic personas as able to “embody” underrepresented demographics or produce “lived experience” insights risk blurring the distinction between simulated text and lived, situated, relational experience. The proposed “structured debate format,” led by a synthetic PPIE Lead persona, also appears to model PPIE as a form of generated feedback rather than a collaborative process involving accountability, relationship-building, and shared decision-making [1].

A further concern is that the paper does not clearly describe substantive roles for human public contributors, PPIE professionals, or community partners in the design, governance, interpretation, or evaluation of Panelyze’s outputs [1]. This matters because the groups named as absent or marginalized in PPIE are not merely data gaps to be simulated. They are people and communities whose involvement requires consent, context, trust, recognition, payment, reciprocity, and influence. Without those conditions, synthetic PPIE may risk minimizing or displacing patient and public voices while appearing to solve the very inequities it is intended to address [1].

Essentially, Steele et al [1] appear to have created a compelling solution to real problems facing PPIE which, at least partially, replaces or minimizes patients’ collaboration in and shaping of health research through lived experience. Instead of providing researchers with the means to learn how to reach those often labeled as “hard to reach,” they have created a compelling generative AI platform, which allows software to speak for humans. It even takes the burden off PPIE Leads by simulating focus group facilitation.

We emphasize that this is not a unique development. PPIE approaches have struggled to achieve genuine collaboration between patients, members of the public, and researchers since their inception [8,80,81]. Since the period when PPIE was referred to as “consumer involvement,” its practitioners have been pressured to create greater value for money, speed, and measurable impact in research [8,80,81]. Some authors have noted a relationship between mandates to do PPIE and PPIE becoming “tokenistic” or a “tick box” exercise [8,80-82]. This refers to doing PPIE primarily to satisfy requirements, rather than to improve research’s relevance to patient communities and their priorities, or to speaking to a person and referring to them as representative of a much wider diversity of experiences, identities, and perspectives [8,80-82]. We remain concerned that increasing pressure for efficiency and cost savings could lead to these tools being used in ways that gradually reduce opportunities for direct engagement with people. We also recognize that researchers and involvement teams are often working under significant resource and time pressures. While these pressures are real, they should encourage new ways of reaching people rather than replacing direct engagement with synthetic alternatives.

Meaningful collaboration with minoritized, underserved, and seldom-heard communities, including people with limited English or low literacy, can be challenging for research teams. The barriers between research institutions and these communities are often substantial, shaped by language, accessibility, trust, time, power, prior experiences of services, and practical constraints. Building relationships and creating inclusive routes into involvement are therefore time and resource-intensive, particularly in research environments where time and resources are often limited. Researchers are also not always trained in the relational, cultural, and facilitation skills needed to do this work well.

Yet, the purpose of PPIE is the collaboration between those conducting research and those affected by it. It has a democratic function—bringing lived experience, community knowledge, and public priorities into research in ways that can shape questions, methods, interpretation, and implementation and ultimately shape the services people use. In this context, using generative AI to consult digital simulations of minoritized peoples based on existing data, instead of talking to minoritized peoples, risks creating an elaborate and compelling route back to tokenism.

Even when used only as a first step, such tools will likely displace the people they claim to represent, at least in part. These tools omit their voices and lived experiences, substituting something else in their place. While data, quotations, or apparent insights that sound like lived experience might shape a project through Panelyze, their voices and lived experiences do not. The data, quotations, and insights that Panelyze helps to produce in the name of their identities can also be biased, distorted, and inaccurate. The risk, then, is that representational absence is treated as a problem of synthetic generation rather than as a problem of power, access, trust, resourcing, and accountability.

Importantly, some PPIE researchers have noted that PPIE Leads are actually the ones who often write the first draft of plain English summaries and similar materials [36]. Panelyze’s use of a virtual PPIE Lead, therefore, suggests that PPIE professionals, as well as public contributors, could be partially omitted or displaced by this new technology. This matters because PPIE work is not only the production of accessible text or feedback; it also involves judgment, facilitation, relationship-building, and ethical care.

### Risk 4: Limited Learning

AI expert Ethan Mollick [54] argues that 2 of the 5 times not to use AI chatbots are when “you need to learn and synthesize new ideas or information” and “when the effort is the point”. PPIE, likewise, is about effort, doing something out of need to learn something, and considering new ideas. Both warnings are directly relevant to PPIE. Good involvement is not simply a route to quicker summaries, sharper plain English text, or more diverse-sounding feedback. It is a process through which researchers learn from people, encounter unfamiliar priorities, disagree, test assumptions, and develop more accountable research practices. If synthetic personas are used to shortcut this process, researchers may obtain polished outputs while losing the difficult but necessary learning that comes from human dialogue, relationship-building, and shared reflection [83,84].

Staley and Barron [85] have even argued that learning is one of the primary outcomes of public involvement. For public contributors, in particular, documented outcomes and impacts of PPIE have included validation, hope, and satisfaction from collaborating on research relevant to their communities, improving confidence, and building relationships with others who have similar experiences [82,86-92]. Patients and members of the public speak from their lived experiences of health, services, institutions, and sociocultural realities. Researchers respond, reflect, and are changed through learning about these experiences. Public collaborators likewise change by learning about and through research and its processes. This dynamic cannot be reduced to simulated interaction. Synthetic personas, by contrast, do not “learn” from their difficulties, exchanges, and successes. They may appear to alter when prompts, model settings, or later versions of LLMs change, but this is not the same as human learning through participation, relationship, and reflection.

Using chatbots or generative AI for synthetic PPIE insights in place of engagement with people goes against the point of PPIE. PPIE is about people and relationships. Human connection is what makes public involvement meaningful. When using LLM chatbots to generate outputs to be used in lieu of the voices of those with lived experience, there is a risk of reducing, displacing, or simulating the very voices PPIE was designed to include. PPIE gives patients a chance to learn about research, influence its direction, and shape work that may affect their treatments, services, communities, and understanding of health. It also gives researchers opportunities to learn from forms of knowledge they may not otherwise encounter, for example, what it feels like to live with or manage a medical condition; the words that people prefer, reject, or find distressing in settings like neonatal intensive care units; what problems people actually want AI to solve in health research; what researchers currently misunderstand about disability; and many other insights that emerge through dialogue, trust, and situated experience [39,93,94].

## Clarifying Generative AI Opportunities

To clarify, we do not outright reject AI in all its forms. AI has the potential to improve health research and patient outcomes in diverse areas. Some applications focus on reducing the time required for research tasks such as screening studies for systematic reviews [95]. Others involve analyzing large, complex datasets to support evaluation, prediction, or detection, including the assessment of cancer treatments’ effectiveness [22] and early disease detection [96]. AI may also support clinical decision-making by analyzing large amounts of case notes to identify loneliness in patients or by reviewing data from wearable technology to improve identification of arrhythmia, cardiovascular disease, or heart failure [97-100].

For PPIE, specifically, carefully governed and ethical generative AI shows some clear opportunities to support PPIE leads and increase access to PPIE and research. We recognize that AI may help some people participate more fully in research and involvement activities, particularly where there are barriers relating to communication, language, disability, geography, or accessibility. However, these benefits should support human involvement rather than replace it. There are online communities where patients can congregate and meet one another, and where organizations for health conditions can do the same—the benefit of AI lies in connecting and linking these different people, communities, and organizations [101-103]. Most striking is generative AI’s ability to support translation between languages almost instantly and to create captions and audio descriptions for disabled collaborators, including Deaf, deaf, hard-of-hearing, blind, or visually impaired people [25,63,99]. Generative AI can also help neurodivergent collaborators in interpreting social context, preparing responses, processing information, or reducing communication demands [42,100]. In these cases, AI can grow and support human connection by showing people where they can connect with one another, instead of replacing or displacing human connection.

## Conclusions

### Summarizing Risks and Cautions

In summary, we acknowledge that generative AI may offer useful support for PPIE, particularly where it improves access, communication, translation, preparation, and connection. However, we have also shown that it carries significant risks when used to simulate or substitute for people with lived experience. These include risks of (1) distortion, bias, and hallucination; (2) opaque data representation and unequal impacts; (3) the minimization or displacement of patient and public voices; and (4) reduced learning for researchers and public contributors. Generative AI should therefore be used cautiously, transparently, and in line with emerging best practice [22,49,98]. Most importantly, it should not replace the relationships, dialogue, and accountability that make PPIE meaningful [25,54,104].

### Recommended Checklist for Valid and Genuine PPIE

To help establish best practices for generative AI in conducting PPIE, we have created a list of recommendations for generative AI, built on existing practical checklists, recommendations, and queries that engage with the risks we outline [25,54,104-106].

- Speaking from experience: When using generative AI, ask yourself, “Could a person speak on this topic or question based on their lived experience?” If yes, ask that question of at least one patient or member of the public with relevant lived experience.
- PPIE insights: Did AI generate the quotations that you are planning to use as insights in a proposal, report, or deliverable? If yes, ask a patient how their experience relates to your project’s questions and the difficulties you might be having.
- Effort: Will struggling with the task at hand make you better at it in the future? Is there a chance that the effort you spend on this task will strengthen your skills? Is there any chance that outsourcing this to AI will weaken your ability to do this over time? If so, try doing this without AI.
- Accessibility: Is there someone in a meeting who needs an AI chatbot for accessibility reasons (ie, summaries to support neurodivergence, closed captions for sensory impairment)? If not, do not use it. If so, do.
- Consent: When using AI bots in meetings, obtain clear consent from all those present for the bots’ participation.
- Reflecting: The time spent reflecting on notes and summarizing meetings can be helpful and bring ideas and insights. Do not completely outsource this to AI.
- New topic: Try generative AI as a type of search function to get you started on something you know nothing about. Ask critical questions and request clear sources and websites to help you learn on your own.
- Transparency: Be clear with professionals, patients, and members of the public about where and how you are using generative AI and where and how you are not.
- Data security: Take responsibility for the safety of the data shared with generative AI platforms. When inputting data into generative AI, make sure to use anonymized data. Use more secure LLMs and chatbots tied to your institution’s data protection practices. At many universities, this is often Microsoft Copilot.
- Human verification: Make sure that the materials you share with others are reviewed and edited by humans before they are shared with others.
- Relationships: When using generative AI in and around PPIE, make sure that relationships with real people remain the focus of your work, as opposed to deliverables—documents, quotations, and bullet points.
- Accessibility: AI can play a valuable role in areas such as accessibility support, transcription, and translation; however, it should only be used to support specific collaborators’ access needs, with full ongoing consent, and never to replace genuine patient perspectives or lived experiences.

### Future Research

As one response to the risks of generative AI in PPIE and collaborative research, several authors have argued that patients, members of the public, and communities should be involved in AI development, implementation, and governance, and that researchers should explore their expectations, concerns, and hopes for AI in health research and care [2,39-42]. However, among these areas, perhaps the least work has been done to codevelop AI with members of the public [41,42]. Future research has the potential to show the impacts that cocreation of AI and its collaborative implementation can have. At minimum, it should therefore examine how participatory and cocreative approaches influence AI design, implementation, acceptability, accountability, and impact. However, as we have shown, more accurate and effective AI chatbots are still categorically incapable of replacing the contributions of people with lived experiences. The greatest question for future research is thus whether AI development processes can genuinely share power, recognize lived experience, exist without replacing lived experiences, and avoid reproducing the same tokenistic practices that have affected PPIE more broadly.

### Signed

Those who support this paper’s argument and have reviewed the final version of this paper but did not contribute as authors are listed below:

- Kim Airey (Project Manager, UCL)
- Katherine Barrett (Public Contributor)
- Helen A. Blake, PhD (Senior Research Fellow, UCL)
- Mr GD Cairns, MBE (Public Contributor)
- Elizabeth Eveleigh, PhD, ANutr (Research Associate, University of Cambridge)
- Alison Finch, MBE, PhD (Assistant Chief Nurse, UCLH)
- Sarah Fisher (Neurodivergent Women’s Health Advocate and PPI Representative)
- Lina Gonzalez, MSc (Research Fellow, UCL)
- Jason Grant (Lived Experience Consultant)
- Christopher Griffiths, DPhil (Professor of Primary Care at QMUL)
- Sarah Griffiths, PhD (Senior Alzheimer’s Society Research Fellow)
- Veenu Gupta, PhD (Lived Experience Researcher)
- Savitri Hensman (Former Patient, Service User, Carer, and Public Involvement Coordinator, ARC South London)
- Alice (Alyson) Hillis, PhD (Senior Research Fellow, City St. George’s)
- Sarah Jasim, PhD (Care Policy and Evaluation Centre, LSE)
- Danielle Lamb, PhD (Senior Researcher, UCL)
- Monica Lakhanpaul, DM (Professor of Integrated Community Child Health; Vice-Provost of Research, Innovation and Global Engagement, UCL)
- Kristin Liabo (Associate Professor, NIHR ARC South West Peninsula)
- Joan Manning (Public Contributor)
- Nikhwat Khan Marawat (Director, The Delicate Mind)
- Rose-Marie McDonald (Public Contributor)
- Sarah Markham, PhD (Public Contributor)
- Raj Mehta (Public Contributor)
- Begonya Nafria (Head - Patient Engagement in Research, Sant Joan de Deu Research Institute)
- Danielle Nimmons (GP and Alzheimer’s Society Clinical Training Fellow, Leeds University)
- Andrea Okoloekwe (Deputy Chief Pharmacist, NHS ELFT)
- Tom Osborn, PhD (Senior Research Fellow at UCL)
- Gita Ramdharry, PhD (Department of Neuromuscular Diseases, UCL)
- Nicola Rushent (Public Contributor)
- Hannah Savage (Research Support Officer, UCL)
- Iris van der Scheer, PhD (Researcher, UCL)
- Nira Shah (Public Contributor)
- Sudhir Shah (Seasoned Public Contributor)
- Jessica Sheringham, PhD (Senior Research Fellow, UCL)
- Margaret Sim (Public Contributor)
- Deb Smith (Public Contributor)
- Charitini Stavropoulou, PhD (Professor, City St. George’s University of London)
- Steph Taylor, PhD (Professor of Public Health and Primary Care, QMUL)
- Jacqueline Walumbe (Advanced Practice Physiotherapist, UCLH)
- Jiunn Wang, PhD (Senior Research Fellow, UCL)
- Andrea Wright, MSc (Advanced Practice Physiotherapist, UCLH)

## Abbreviations

ARC: Applied Research Collaboration
ICE: Immigration and Customs Enforcement
LLM: large language model
MIPP: Maximising Impact and Public Partnerships
NHS: National Health Service
NIHR: National Institute for Health and Care Research
PIC: Public Involvement Community
PPIE: patient and public involvement and engagement
RAG: retrieval-augmented generation
SIRO: Senior Information Risk Owner
